# Plant and Mammal Aquaporins: Same but Different

**DOI:** 10.3390/ijms19020521

**Published:** 2018-02-08

**Authors:** Timothée Laloux, Bruna Junqueira, Laurie C. Maistriaux, Jahed Ahmed, Agnieszka Jurkiewicz, François Chaumont

**Affiliations:** Institut des Sciences de la Vie, Université catholique de Louvain, Croix du Sud 4-L7.07.14, B-1348 Louvain-la Neuve, Belgium; timothee.laloux@uclouvain.be (T.L.); bruna.teodoro@uclouvain.be (B.J.); laurie.maistriaux@uclouvain.be (L.C.M.); jahed.ahmed@uclouvain.be (J.A.); agnieszka.jurkiewicz@uclouvain.be (A.J.)

**Keywords:** aquaporins, plant, mammal, regulation, heteromerization, specificity, gating

## Abstract

Aquaporins (AQPs) constitute an ancient and diverse protein family present in all living organisms, indicating a common ancient ancestor. However, during evolution, these organisms appear and evolve differently, leading to different cell organizations and physiological processes. Amongst the eukaryotes, an important distinction between plants and animals is evident, the most conspicuous difference being that plants are sessile organisms facing ever-changing environmental conditions. In addition, plants are mostly autotrophic, being able to synthesize carbohydrates molecules from the carbon dioxide in the air during the process of photosynthesis, using sunlight as an energy source. It is therefore interesting to analyze how, in these different contexts specific to both kingdoms of life, AQP function and regulation evolved. This review aims at highlighting similarities and differences between plant and mammal AQPs. Emphasis is given to the comparison of isoform numbers, their substrate selectivity, the regulation of the subcellular localization, and the channel activity.

## 1. Introduction

Membrane intrinsic proteins (MIPs), also ambiguously named aquaporins (AQPs), are channel proteins facilitating the passive movement of water and an increasing list of small solutes across biological membranes. MIPs constitute an ancient family of proteins, present in all three kingdoms of life (Eukarya, Bacteria, and Archaea), suggesting their essential role in basal life functions. MIPs are small proteins (21–35 kDa) comprised of six transmembrane α-helices (TM) connected by five loops as well as cytosol-facing N- and C-termini. Two additional short α-helices coming from loops B and E dip halfway into the membrane from opposite sides [1]. The symmetrical structure of the MIPs probably arose from the duplication and inversion of a half-size gene encoding for three transmembrane helix motifs [2,3]. The eight membrane-embedded domains form a pore with two selectivity filters allowing charge and size exclusion of the entering molecules. A pair of NPA (Asn-Pro-Ala) motifs held by the diving short helices constitutes the first filter region. They position entering molecules correctly by forming hydrogen bonds and establishing an electrostatic repulsion of cations and protons [4,5]. The second filter, the ar/R (aromatic/arginine) region, located at the extracellular portion of the channel, creates the narrowest section of the pore [1]. MIPs associate as tetramers in membranes, each monomer being an independent channel. Interestingly, while homotetramers are mostly observed in mammals, the association of AQPs in heterotetramers has been thoroughly described in plant species. The tetrameric structure also suggests the presence of a fifth pore in its center, whose presence and role in solute transport is still a matter of debate. Despite those shared structural features, the MIP family is a large and diversified protein family at the sequence and functional levels. A major distinction is attributed to a putative ancient split of the MIP family between the water channels and glycerol facilitators (aka AQP per se and aquaglyceroporins (GLPs)). The traditional view is that both families then expanded and diversified in multicellular organisms due to rounds of gene/genome duplications and horizontal gene transfer (HGT) events. In comparison with mammals, plants express a much greater number of MIPs that were probably conserved due to the functional advantages linked to the plant lifestyle.

## 2. Diversity and Phylogeny

Mammals usually possess 12 to 15 isoforms gathered into 13 subfamilies (AQP0–12). Humans differ by displaying 18 paralogs due to tandem duplications leading to four additional AQP7 pseudogenes and a second copy of AQP12. This classical view recently evolved as older lineages of mammals (Metatheria and Prototheria) were shown to retain additional classes (AQP13–14) [6]. Animal MIPs are usually classified into four groups: the classical or orthodox AQPs (AQP0, 1, 2, 4, 5, 6, 14) that are associated with water transport, the aqua-ammoniaporins (AQP8) sometimes included in the orthodox AQPs, the aquaglyceroporins (AQP3, 7, 9, 10, 13), and the unorthodox AQPs (also called superaquaporins) (AQP11–12) [6,7,8,9]. The latter ones have low homology with the other mammal AQPs and harbor an unusual N-terminal NPA motif (NPC for AQP11 and NPT for AQP12), the C-terminal NPA motif being conserved [10]. Unlike this relatively stable state shared by mammals, plants show a much greater diversity. They usually possess far more isoforms, and their number is species-dependent. This is probably largely due to the greater duplication rates of plant genomes but also to the adaptation potential brought by AQPs, especially relevant in view of the static lifestyle of plants. To date, 35 isoforms were found in the model species *Arabidopsis thaliana* [11,12], 38 in *Zea mays* [13], and 71 in upland cotton (*Gossypium hirsutun*) [14]. The greatest number is currently found in oil seed rape *Brassica napus*, displaying 121 full-length AQPs [15]. Plants MIPs are traditionally classified into seven subfamilies, named from their subcellular localization and other specific features. Five of these subfamilies are found in seed plants: plasma membrane intrinsic proteins (PIPs), tonoplast intrinsic proteins (TIPs), NOD26-like intrinsic proteins (NIPs), small basic intrinsic proteins (SIPs), and unknown intrinsic proteins (XIP) that have not been detected in monocots and Brassicaceae so far [16]. Hybrid intrinsic proteins (HIPs) and GLpF-like intrinsic proteins (GIPs) are only found in older lineages (mosses), suggesting a loss of these between the ancestor of the vascular and seed plants [8]. It is noteworthy that GIPs are the only GLPs found in plants. Their acquisition by HGT from bacteria has been suggested [17], and their loss could have been caused by redundancy with the already present glycerol-transporting NIPs [18]. Furthermore, the origin of NIPs, either as an ancestral family [7,19] or acquired HGT either from bacterial AQP-Z [20] or other origin, is still questioned [6,7,8]. Also, whether the ability of NIPs to transport glycerol comes from their ancestor or was acquired by co-option in plants after the HGT event is still unknown [8]. Interestingly, green algae show PIPs and GIPs, as well as other five families (MIP A–E) that are not present in any plant lineage [21]. Finally, a unique family, comprising the large intrinsic proteins (LIPs) was recently discovered in diatoms [22]. These subfamilies are additionally divided into paralog groups. The PIP subfamily, for instance, is divided into PIP1 and PIP2 based on their sequence homology [13]. This correlates with their different ability to reach the plasma membrane and act as active channels [23]. Each plant MIP is usually named with the abbreviation of its subfamily, followed by two numbers translating their phylogenetic group and timing of discoveries, respectively. Unlike the mammal AQPs, the nomenclature of plant AQPs leads to confusion, as two proteins with the same name, coming from two different species, are not necessarily orthologues. Furthermore, different phylogenetic analyses producing contrasting results are rendering the paralog group classification inconsistent [8]. Recently, a phylogenetic study defined 19 clusters of orthologous genes for flowering plants, with, for instance, the PIP family being further divided into three clusters instead of two [7]. This classification agrees with the former classification and nomenclature of *A. thaliana* [11,24]. This illustrates the need for a global nomenclature, based on the evolutionary framework. Then, nomenclature could help bring the two worlds of animal and plant MIPs closer together. Indeed, a phylogenetic study restricted to proteins with high (>25%) amino acid identity showed the closeness between the four groups of animal MIPs and the plant subfamilies (PIP, TIP, NIP, and SIP), suggesting a new paradigm of vertical transfer of four ancestral subfamilies [7]. These four families, A, B, C, and D, gather respectively AQP1-like and PIP, AQP8-like and TIP, AQP3-like and NIP, and finally AQP11-like and the SIP subfamilies [7]. The relevance of this gathering to the functional level is consistent to some extent [7,19] and induces predictions about substrate specificity, for instance, that can be easily verified. However, the evolutionary pathway of the MIP family is far from being resolved because of blank spaces that need to be filled in and contrasting results arising from sequence selection criteria and methods used to perform the phylogenetic analyses. From here, the term AQP will be used in a general way as a synonym of MIP.

## 3. Specificity and Physiological Roles

In addition to water, AQPs are involved in the transport of small solutes such as glycerol, urea, ammonia, H_2_O_2_, CO_2_, O_2_, boron, arsenic, antimony, silicon, sodium ion, and others (Table 1) [25,26,27,28,29]. With such a basic function as transporting water and solutes along their gradient, AQPs are involved in numerous and diverse physiological functions. In this section, several functions linked to the AQPs transport capacity will be described, but it is noteworthy that AQPs could have acquired additional specific roles. For instance, AQP0 is involved in cell junctions, a role specific to animals [30,31].

### 3.1. Water

AQPs are important players of water homeostasis at the cell, organ, and organism levels. Even though a complete overview of each mammal or plant AQP expression profile and their different roles is far beyond the scope of this review, it is noteworthy that AQPs are highly expressed in all fluid-related structures. Several AQPs are expressed and play important roles in human kidneys (reviewed by Noda et al. [32]), the obvious key players of mammalian water homeostasis, but also in salivary and sweat glands (reviewed by Nejsum et al. [33]), pancreatic cells (reviewed by Delporte, [34]), and lacrimal glands (reviewed by Schey et al. [35]). The involvement of AQPs in plant water homeostasis is also compelling [36,37], in a completely different way. Unlike animals, plants are autotrophic. Via the photosynthetic process, plants are able to synthesize sugar molecules from carbon dioxide and water using light energy. As the major source of CO_2_ is atmospheric, plants face the necessity of having a constant exchange of gases with the outside, occurring through stomata located mainly on leaves. These are multicellular structures working as inflatable doors whose opening can be modulated in adverse conditions. However, in order to survive, plants have to cope with the loss of water (called transpiration) that is inevitably associated with CO_2_ intake. Therefore, water is constantly taken up by the roots and transported across the plant to the leaves, where it is evaporated due to a gradient of negative pressure (or tension). This long-distance vertical transport occurs across the vascular tissues, the xylem vessels consisting mostly in low-resistance dead cells. However, short-distance radial movement across the root and the leaf requires the involvement of the living tissues. Water crosses plant tissues using several pathways: the apoplastic path (within the cell wall), the symplastic path (across the plasmodesmata, which are connections across the cell wall, allowing a cytoplasmic continuity between cells), or the transcellular path (requiring water to cross cell membranes). AQPs come into play in the latter case by regulating membrane permeability [36,38]. PIPs and TIPs are probably the most important players regarding water homeostasis in plants thanks to their high-water transport capacity. Plant reproduction also requires some structures with specific hydraulic properties. Several studies have shown the involvement of AQPs in anther dehydration [39], pollen development and germination [40,41], and seed germination in several species [42,43,44].

In addition, AQPs located in the plasma membrane and tonoplast probably have an important role in plant cell growth and organ development [45,46,47]. Indeed, plant cells grow thanks to the turgescence, meaning the entry and storage of water in large vacuoles. The final size of the cell is obtained when the pressure from the water-filled vacuole equilibrates with the cell wall rigidity. Analogously, such hydrodynamics can be relevant for cell migration and movement in mammals.

Indeed, it has been suggested that the polarized transmembrane water flow modulated by AQPs facilitates cell volume and shape changes as well as propulsion. The role of AQPs was demonstrated in different migration mechanisms either dependent of cytoskeleton remodeling (actin polymerization and myosin II-mediated contractility) or not (water permeation only, mediated by polarized localization of ion pumps and AQPs, “osmotic engine model”) [121]. The large number of reports showing the implication of various AQPs in the migration of diverse cell types suggests the wide conservation of this first-unsuspected role of mammal AQPs (reviewed by Papadopoulos, M.C., Saadoun, S. & Verkman, 2008 [122]). Such a role implies many therapeutic interests (relying on either improved or repressed cell migration), including the reduction of tumor spread. Beyond facilitated motility, the involvement of AQPs in tumor cell physiology is more and more under the spotlight, as they have been repeatedly shown to be associated with tumor angiogenesis, metabolism, and grade of numerous types of cancer (for more information, see Verkman et al. [123], Papadopoulos & Saadoun [124], and Wang et al. [125]). The recurrent implication of AQPs in cancers and other multiple diseases turned them into key targets for drug development strategies. Such interest for specific regulators and inhibitors is also increasing in the plant biology field for scientific purposes.

### 3.2. Hydrogen Peroxide

Hydrogen peroxide is a signaling molecule regulating a wide range of metabolic processes in all living organisms [126]. Intracellular levels of H_2_O_2_ must be tightly regulated because it can oxidize various cellular targets that actually cause oxidative damage or apoptosis. Currently, it is an established fact that AQPs are involved in transmembrane diffusion of H_2_O_2_ [53,127]. Although H_2_O_2_ is involved in cytotoxicity and senescence in plants [126], it is also involved in signal transduction as a second messenger, as well as in the biosynthesis and development-related modifications of structural components of cell walls [128]. Several PIPs, TIPs, and XIPs have been shown to transport H_2_O_2_ when expressed in yeast (Table 1) [52,57,129]. AtPIP1;4 facilitates the cellular entry of apoplastic H_2_O_2_ produced after infection with a bacterial pathogen [130], and AtPIP2;1 facilitates H_2_O_2_ entry into guard cells, leading to stomatal closure triggered by abscisic acid or the pathogen associated molecular pattern flg22 [131]. In mammals, AQP3, 8, and 9 are also capable of facilitating the transmembrane diffusion of H_2_O_2_ [132,133,134,135]. AQP3 and AQP8 facilitated H_2_O_2_ diffusion is required for NF-κB activation in keratinocytes in the development of psoriasis or in response to environmental stresses in colonic epithelia and teleost spermatozoon motility [112,136,137].

### 3.3. Ammonia and Urea

Nitrogen is one of the constituents of amino acid, which is essential for all living organisms. Ammonia and urea are broadly used as nitrogen fertilizers for plants and are also the byproducts of many metabolic processes [138]. Several TIPs showing divergence in the ar/R selectivity filter facilitate ammonia diffusion (Table 1) [19,139]. The structure of *Arabidopsis* TIP2;1 reveals an extended selectivity filter region with a relatively wide pore and polar nature explaining the ammonia permeability [140]. Interestingly, the identified determinants in the extended selectivity filter are sufficient to convert the water-specific human AQP1 into an TIP2;1-like ammonia channel. The mammalian AQP3, AQP6, AQP7, AQP8, AQP9, and, possibly, AQP10 are also permeable to ammonia (Table 1) [9,141]. The ammonia-permeable AQPs are associated with nitrogen homeostasis and contribute to the intracellular acid-base homeostasis [142]. On the other hand, AQP-mediated urea conductance in plants might contribute to the detoxification of urea excess and facilitate plant organ development [54,129,143]. Indeed, a few NIPs and PIPs have been reported to facilitate urea transmembrane diffusion in plants (Table 1) [144,145]. In mammals, AQP3, AQP6, AQP7, AQP9, and AQP10 also facilitate urea diffusion (Table 1) [141] and are involved in energy metabolism and epidermal hydration, but the exact physiological roles of most aquaglyceroporins in diffusing urea are not yet understood [142].

### 3.4. Metalloids

The metalloids are a group of physiologically important elements whose physical and chemical properties describe them as being neither metals nor nonmetals, some of which are essential or at least beneficial for plant growth (boron and silicon) while others are toxic (arsenic and antimony). The ability to AQPs to facilitate the diffusion of the trivalent arsenite (structurally similar to glycerol) could be an ancestral feature in plants, as several members of NIPs, XIPs, and few homologs of PIPs share this capacity [50,71] (Table 1). This is an important health issue as arsenic is a food chain contaminant [146]. Mammalian AQPs (AQP3, AQP7, AQP9, and AQP10) are also able to facilitate the transmembrane diffusion of arsenite [147]. Arsenite containing compounds are toxic to cells, yet this metalloid is used as a chemotherapeutic agent for treating acute promyelocytic leukemia and diseases caused by protozoan parasites [148].

Phytotoxic antimony is transported through AQPs (plant NIPs and the mammalian aquaglyceroporins AQP3, AQP7, AQP9, and AQP10) in the form of antimonite, a toxic element that can also enter into the food chain (Table 1) [25,139]. The greater expression of AQP3, AQP7, and AQP9 render human leukemia cells as well as lung adenocarcinoma cells hypersensitive to the antimonite due to greater levels of accumulation [149,150]. Antimonite is used to treat leishmaniosis, a disease caused by the parasite *Leishmania* spp. Disruption of one of the aquaglyceroporin alleles in *Leishmania major* led the parasite to be tenfold more resistant to treatment with antimonite [151]. Antimony is used to treat some other parasitic infections and some types of leukemia. The discovery of the roles of AQPs in antimonite transport can lead in the future to therapeutic or toxicological applications.

Boron is an essential element for the plant cell wall and structure. Boric acid has a dimension similar to urea and was shown to be transported by several NIPs, XIPs, and, more rarely, PIPs (Table 1) [69,152,153,154]. Up to now, there are no reports demonstrating that mammalian AQPs can permeate boron.

Silicon, commonly absorbed from the soil as silicic acid, is one of the beneficial elements for plant growth and protection from numerous biotic and abiotic stresses [19]. Plant silicon transporters belonging to the NIPs have been reported in many higher plants (Table 1) [155,156,157]. Silicon is also one of the abundant and differentially distributed trace elements in mammals, where it might play essential biological functions [27]. Silicon concentrations in body fluids might be regulated by AQP-mediated silicon permeation at the surface of different cell types. Indeed, the human aquaglyceroporins AQP3, AQP7, AQP9, and AQP10 are able to act as silicon channels (Table 1) [27].

### 3.5. Gases

The first evidence of facilitated diffusion of CO_2_ across membranes came from research on the human AQP1 [78]. Since then, other studies reported that mammal AQP0, AQP4, AQP5 and AQP6 are also involved in CO_2_ diffusion (Table 1). Atomic molecular dynamic simulation data based on mammal AQP1 crystal structures show that AQP1-mediated CO_2_ diffusion can be expected in membranes with low intrinsic CO_2_ permeability [158]. However, it is more likely that CO_2_ diffusion is mediated through the central pore formed by the tetramer, because it is lined by hydrophobic amino acid residues and therefore is an ideal path for hydrophobic CO_2_ molecules [159]. In plants, the rate of CO_2_ conductance is the limiting factor for photosynthesis. Plasma membrane AQPs belonging to the PIP1 subfamily have been shown to facilitate CO_2_ membrane diffusion (Table 1) [19,50,52,160,161,162]. For instance, reducing or overexpressing the tobacco AQP1 expression level in plants results in a reduction or increase of the photosynthesis rate, respectively [51]. Recently, two AQPs (human AQP1 and tobacco PIP1;3) were shown to most likely act as oxygen (O_2_) channels when expressed in yeast protoplasts [163]. Finally, AQP1 has also been reported to facilitate transmembrane diffusion of nitric oxide [81].

### 3.6. Ions

The heterologous expression in *Xenopus* oocytes of human AQP1 led to a PKA-activated ion permeability probably mediated by phosphorylation of AQP1 [164] and a cGMP-activated ion permeability via direct binding of cGMP to the C-terminus of AQP1 [165]. AQP1 can therefore act as an ion channel, with the ion pathway probably residing in the central pore formed by the tetrameric assembly. Whether this ion conductance has a physiological relevance remains an open question. However, dual water:ion movement is vital for several pathophysiological processes including tumour angiogenesis, cell migration [166], and epilepsy [167], so there is a possibility that AQPs perform this dual function. The pH-sensitive ion permeability of AQP6 was also demonstrated [168]. In this case, it is proposed that the monomeric pore is the ion pathway, and assumed that a point mutation to a pore-lining residue of AQP6 abolished the ion permeability [105]. In plants, there is evidence that NIP can also act as a voltage-gated, anion-biased ion channel [169,170]. Recently, the discovery of a non-selective cation channel activity for the *Arabidopsis* PIP2;1 when expressed in *Xenopus* oocytes or *Saccharomyces cerevisiae* [28] led to the hypothesis that AQPs could play a role in cell ion homeostasis and also drive water transport in the absence of water potential differences using Na^+^ and water co-transport.

## 4. Regulation Mechanisms

AQPs, as membrane integral proteins, are synthesized and co-translationally inserted in the endoplasmic reticulum (ER) membrane and travel across the secretory pathway to reach their target membrane. There, their activity can be tightly modulated by opening or closing the channels (gating) in order to control the water/solute homeostasis of cells [36]. All these processes are subject to various regulation mechanisms, several of them being described below. However, other mechanisms will surely be discovered in the future years, due to the extensive list of AQP interactants identified in different large scale proteomic and interatomic studies (i.e., Bellati et al. [171]).

### 4.1. Trafficking

AQPs are present in most biological membranes. Plant PIPs and most mammal AQPs usually localize in the plasma membrane, with a polarized localization in numerous cases (for a complete recent review about plant and mammal AQPs, see Chevalier and Chaumont [172] and Li and Wang [173]). Some AQPs have internal localization, such as the TIPs, which are found in the vacuolar membrane. Mammal superaquaporins and plant SIPs are also found in internal membranes. SIPs and AQP11 remain mainly in the ER [69,174], while the precise intracellular localization of AQP12 is not determined [175]. While NIPs were first discovered in the peribacteroid membrane of a nitrogen-fixing symbiosome of soybean [62] and other legume species [176,177], they are mostly present in the plasma membrane. Some mammal AQPs are present in intracellular vesicles for functional or storage purposes. While AQP6 seems to have only intracellular functions [107,178], AQP2 shuttles from intracellular vesicles to the plasma membrane of the principal cells of renal collecting ducts under a vasopressin-triggered cascade [179,180]. AQP8 localizes mainly in the intracellular vesicles in rat hepatocytes, while its relocalization to the plasma membrane was stimulated by cAMP [181]. AQP8 and AQP9 were also detected in the inner mitochondrial membrane [182,183,184]. Similarly, TIP5;1 from *Arabidopsis* was detected in the mitochondria when expressed under the control of the constitutive CaMV-35S promoter [185], but this data was not confirmed when its endogenous promoter was used [40]. Finally, tobacco AQP1, belonging to the PIP1 group, was detected both in the plasma membrane and the inner membrane of chloroplasts, where photosynthesis takes place [186].

AQP trafficking regulation acts at two sequential steps: targeting to their resident membrane and removal from the membrane for degradation or recycling in the endosome. These processes are very dynamic, and since this was the first evidence showing modification of AQP2 subcellular localization triggered by vasopressin [179,180], the short-term subcellular regulation of AQPs has become the focus of many studies. In order to fine tune the membrane water flow, cells can indeed internalize AQPs or relocate them from endomembrane compartments to the plasma membrane, highlighting the importance of AQP trafficking in the short-term regulation in response to external stimuli such as environmental stimuli, circadian clock, hormones, and other signaling molecules. We will pinpoint/compare below several mechanisms that control plasma membrane localization of mammal and plant AQPs.

#### 4.1.1. Motifs

Diacidic motifs ([D/E]x[D/E]) are involved in protein ER sorting in both plants and mammals through interaction with the COPII machinery [187]. Mutation of this signal in plant PIPs results in protein ER retention, demonstrating that the presence of the diacidic motif is required for proper plasma membrane targeting [188,189]. However, other ER exit motif(s) should be present in PIPs, as several isoforms that do not contain such diacidic motifs are still able to reach the plasma membrane. On the other hand, adding a diacidic motif to maize PIP1;2, an isoform that, when expressed alone, is retained in the ER, is not sufficient to bring it to the plasma membrane [188]. A new LxxxA motif located in the TM3 was identified to mediate maize PIP2;5 export from the ER to the plasma membrane [190,191]. Interestingly, adding both the diacidic and LxxxA motifs into PIP1;2 was not sufficient to direct the protein to the plasma membrane, suggesting that an ER retention motif is still present and dominant in PIP1;2 [190]. The presence of diacidic and LxxxA motifs in mammal AQP trafficking has never been studied to date. Putative diacidic motifs are found in the C-terminus of human AQP1 to AQP6, AQP9, and AQP10, the N-terminus of AQP8, or at the junction between the TM4 and loop D of AQP7. Interestingly, some human congenital disorders leading to nephrogenic diabetes insipidus (NDI) are caused by AQP2 mistargeting (for review, see Bichet 2012 [192] or Loonen 2008 [193]). Among them, some are retained in the ER and could be linked to a misfolding due to the mutation. On the other hand, other mutations lead to functional isoforms that failed to reach the plasma membrane under vasopressin stimulation or are mistargeted [194,195,196]. Mutations in motifs involved in trafficking regulation could be an explanation for the mistargeting of these mutated versions. However, none of them is associated with putative diacidic motifs.

In mammals, the targeting of the AQP4, the predominant isoform in the brain, relies on two motifs located in the C-terminus: a tyrosine motif (YxxΦ) and a dileucine-like motif [87], both having a role in basolateral membrane targeting [197,198]. Upon substitution of any of the two motifs in alanine, AQP4 was rerouted to the apical membrane instead of the basolateral membrane. The significance of both the tyrosine and dileucine motifs was further confirmed by studying the behavior of a mutated version of AQP2, AQP2-insA [196]. In kidney collecting duct principal cells, AQP2 is mostly found in recycling endosomes, while upon vasopressin stimulation, it is relocalized to the apical membrane [179,180]. Due to a frame shift in the *AQP2* gene, AQP2-insA possesses an extended C-terminus and is targeted to the basolateral membrane under forskolin treatment rather than to the apical membrane as observed for WT AQP2. Kamsteeg et al. [196] revealed that both the leucine-based and tyrosine motifs were added to in the sequence, explaining the new targeting profile.

AQP3 is constitutively targeted to the basolateral membrane of kidney collecting duct principal cells. This localization relies on an N-terminal YRLL motif mixing both a tyrosine and a dileucine motif [199]. Interestingly, when this N-terminus is swapped in AQP2, the AQP2 chimera is relocated into the basolateral membrane, demonstrating the role of both motifs in proper AQP basolateral targeting. To date, no tyrosine or dileucine like motifs have been characterized in plant AQPs.

#### 4.1.2. Phosphorylation

AQP phosphorylation is a well-studied process involved in both gating (see below) and trafficking [173,200,201]. In mammals, the most studied case is AQP2 phosphorylation in kidney collecting duct principal cells. Indeed, the AQP2 relocalization process under vasopressin stimulation involves the phosphorylation status of various serine residues at the C-terminus. The exact role of each serine is still not totally elucidated, but phosphorylation of the Ser256 was shown to be necessary for the proper translocation to the plasma membrane [202,203], while phosphorylation of the Ser264 and Ser269 are not strictly required [204,205]. On the other hand, Ser261 phosphorylation is found in vesicle-resident AQP2 and must be dephosphorylated for plasma membrane translocation [206,207,208]. The trafficking of other AQPs, such as AQP0 [209], AQP1 [210], AQP5 [211], and AQP9 [212], have also been shown to be regulated by phosphorylation, usually promoting plasma membrane targeting.

Many plant AQPs are also phosphorylated at either the N- or C-termini or the loop B, altering their gating and/or their trafficking [213,214,215,216]. For instance, phosphorylation of the Ser283 in the C-terminus of *Arabidopsis* PIP2;1 is required for its trafficking to the plasma membrane in resting conditions [217]. Moreover, the phosphorylation status of Ser283 is involved in the regulation of salt- or H_2_O_2_-induced AtPIP2;1 internalization [217].

#### 4.1.3. Soluble *N*-Ethylmaleimide-Sensitive Factor Protein Attachment Protein Receptor (SNAREs)

In plants, recent studies have highlighted the role of SNARE proteins in PIP subcellular trafficking and regulation [218,219]. The SNARE protein family is conserved among all eukaryotic cells and are involved in vesicle fusion with membranes [220]. SYP121 is a plasma membrane Qa-SNARE [221] that regulates proper trafficking of maize PIP2;5 and *Arabidopsis* PIP2;7 through physical interaction. Expression of a SYP121 dominant negative mutant, the so-called SP2 fragment [222], reduces the amount of PIP in the plasma membrane and, consequently, the cell hydraulic conductivity [218,219]. Another trans-Golgi-localized Qc-SNARE, SYP61 has the same impact on PIP2;7 trafficking [219].

In mammals, SNAREs, as well as other members of the trafficking machinery, are involved in AQP trafficking but, to date, no direct physical interaction has been shown in contrast to what is observed for PIPs in plants. AQP2 colocalized with many SNAREs such as syntaxin-4, syntaxin-3, synaptosomal-associated protein (SNAP) 23, and SNAP25, vesicle-associated membrane proteins (VAMP) 2, VAMP3, and VAMP8 [223,224,225,226], and its proper targeting to the plasma membrane, after vasopressin stimulation, relies on vesicles bearing VAMP2. Indeed, under tetanus toxin treatment, AQP2 is retained in vesicles due to VAMP2 cleavage [227]. AQP5 is also redistributed under the influence of botulinum toxin type A and the subsequent SNAP25 cleavage [228].

Interestingly, SNAREs interact with and modulate both potassium and calcium channel activities in mammals and plants [229,230,231,232,233,234,235,236]. For instance, plant SYP121 physically interacts with the K+ channels AKT1/KC1 to control their trafficking and activity. Altogether, these data suggest that the SNAREs, by controlling AQP and potassium or calcium channel abundance and/or activity in the plasma membrane, act as a coordinator of ion and water uptake to regulate cell expansion.

#### 4.1.4. Ubiquitination

Recycling of proteins is a vital process for cells, and AQPs have been shown to be ubiquitinated in both mammals and plants. AQP1 is ubiquitinated to control its degradation and, hence, to regulate the water influx under hypertonic or hypotonic conditions [237]. AQP2 is also ubiquitinated at the Lys270, a process that, together with phosphorylation, triggers its internalization in kidney collecting duct principal cells [238]. In plants, overexpression of a homolog of the RING membrane-anchor1 E3 ubiquitin ligase decrease the abundance of *Arabidopsis* PIP2;1 in the plasma membrane through a degradation process [239].

#### 4.1.5. Tryptophan-Rich Sensory Protein/Translocator (TSPO)

The *Arabidopsis* TSPO multi-stress regulator also modulates the accumulation of PIP2;7 in the plasma membrane through a physical interaction and its degradation through the autophagic pathway. Interestingly, TSPO in *Arabidopsis* is induced by abiotic stresses, suggesting a new mechanism for AQP abundance regulation in such conditions [240]. The exact role of TSPO homologs in mammals is still today a matter of debate [241] and is, so far, not linked to AQP regulation.

#### 4.1.6. Clathrin

Clathrin-dependent internalization of plant AQPs is highlighted using the drug tyrphostin A23, a well-known inhibitor of clathrin internalization in plants [242]. Indeed, various studies revealed that upon tyrphostin A23 treatment, AQPs are stuck at the plasma membrane and not internalized upon stimuli known to trigger AQP internalization such as NaCl or H_2_O_2_ [243,244]. In mammals, AQP4 directly interacts with a clathrin adaptor through the same motif involved in basolateral targeting (see above) to modulate its endocytosis and targeting to the lysosome [245]. Likewise, AQP2 is concentrated in a clathrin-coated pit and the use of a dominant-negative dynamin blocks AQP2 internalization, demonstrating that mammal AQP abundance in the plasma membrane is also dependent on clathrin-dependent internalization [246].

#### 4.1.7. Lysosomal Trafficking Regulator-Interacting Protein 5 (LIP5)

LIP5 is another known AQP2 regulator [247,248]. LIP5 is involved in multi-vesicular body (MVB) formation and binds MVB cargo proteins [249,250]. LIP5 interacts with the C-terminal tail of AQP2 but does not interact with AQP3 and AQP4. Van Balkom and colleagues [247] later showed that, in LIP5 KO mouse renal cells, AQP2 degradation is reduced by a factor two. Finally, LIP5 binds to the C-terminal MIM1 (MIT-interacting motif 1) of AQP2 preferentially when the Ser264 is phosphorylated [248]. No direct link between the LIP5 homolog in *Arabidopsis* and AQP has been proven so far. Nevertheless, LIP5 was recently shown to be involved in drought stress resistance and abscisic acid response [251] and is also known to regulate plant AQPs.

### 4.2. Heterotetramerization, a New Paradigm for AQP Regulation

AQPs assemble as tetramers, in which one monomer interacts physically with two adjacent monomers. However, they do not build one common channel, as it is the case, for instance, for the voltage-gated K+ channels, but each monomer of the tetramer forms an active pore. Homotetramer assembly was observed in all the AQP structures elucidated so far [31,140,216,252,253,254,255,256,257,258,259,260,261,262,263]. Nevertheless, evidence of the association of different AQP monomers in heterotetramers has been obtained in plants, constituting a milestone in the field of AQP regulation. The first indication of AQP heteromerization came from Harvengt et al. [264], who showed that two TIP isoforms from lentil protein storage vacuoles form hetero-oligomers after cross-linking experiments. The formation of hetero-oligomers composed of maize PIP1 and PIP2 was then demonstrated after their expression in *Xenopus* oocytes [48]. While the expression of PIP2;5 results in an increase in the cell membrane osmotic water permeability coefficient (P_f_), the expression of PIP1;2 does not, as a very low amount of PIP1;2 is detected in the plasma membrane [48]. However, when PIP1;2 is co-expressed with PIP2;5, both PIPs physically interact resulting in a high amount of PIP1;2 in the plasma membrane and a significant increase in P_f_, compared with oocytes expressing PIP2;5 alone, which depends on the quantity of PIP1;2 cRNA injected [48]. Since this discovery, a similar synergistic effect was demonstrated between PIP1s and PIP2s from many other plant species, including beets, mimosa, strawberry, tobacco, rice, and grapevine [265,266,267,268]. When expressed in maize protoplasts, PIP1s are retained in the ER, while PIP2s are targeted to the plasma membrane [23]. However, upon co-expression, PIP1s physically interact with PIP2s, resulting in their relocalization from the ER to the plasma membrane [23]. Actually, PIP2 proteins, but not PIP1s, possess signals that allow them to reach the plasma membrane, and hetero-oligomerization of both proteins is required for plasma membrane targeting of PIP1s (see above) [188,190].

Other experimental data identified a conserved cysteine residue located in the extra-cytosolic loop A of PIPs which is involved in disulfide bridge formation resulting in dimerization of two monomers, suggesting that a tetramer is composed of two pre-assembled dimers [269]. However, this disulfide bond is not required for PIP tetramer assembly, trafficking, and activity in oocytes or plant cells under the tested conditions [269].

The striking evidence of the bona fide hetero-oligomers generated by two different PIP isoforms has been shown by Berny et al. [270]. Nickel affinity chromatography purification of maize PIP1;2 and PIP2;5 isoforms proved that they oligomerize as heterotetramers with three different monomer stoichiometries (1:3, 2:2 and 3:1). Subsequently, in silico 3D modeling of PIP1;2 and PIP2;5 heterotetramers was carried out and highlighted the putative interacting residues in all the transmembrane domains [270]. Single mutation of some of them had a significant impact on the water channel activity of the homo- and/or hetero-tetramers, as well as the protein subcellular localization and/or expression level.

As mentioned above, PIP1-PIP2 heteromerization enhances the membrane permeability [48,271]. In addition, the stoichiometry of the heterotetramers might also influence the substrate specificity of the complexes, as exemplified by the water versus CO_2_ permeability of tobacco AQP1 and PIP2;1 heterotetramers [272]. While the presence of a single PIP2;1 protein within a heterotetramer allows for an increase in P_f_, an increase in the CO_2_ diffusion requires the presence of at least three AQP1 [272].

It is also possible that plant AQPs belonging to different subfamilies interact. Indeed, Murozuka et al. [273] showed interactions between *Arabidopsis* TIPs and a PIP2 isoform when expressed in yeast, using bimolecular fluorescence complementation assays. The physiological relevance of such interaction remains an open question.

AQP hetero-oligomerization in mammals is mainly observed between variants/mutants of monomers encoded by a similar gene. Co-immunoprecipitation of mutated AQP2 (763–772del) with WT AQP2 demonstrated their ability to interact and build so-called hetero-oligomers [274]. Heteromerization of WT AQP2 can reduce the deleterious impact of recessive mutations (recAQP2) responsible for NDI [275]. recAQP2 is believed to possess an abnormal folding impeding the proper oligomerization and, therefore, preventing its targeting to the plasma membrane or leading to its degradation. The presence of a single monomer of WT AQP2 was sufficient to recover adequate function of the tetramer and to allow the proper plasma membrane targeting of the recAQP2 monomers. This finding opens to the possibility of naturally existing wild-type/recessive heterotetramers and their individual contribution to a less severe phenotype. On the other hand, the assembly of WT AQP2/AQP2-insA complexes (mutation in AQP2 allele with an insertion of an adenosine causing a COOH terminal frame shift) leads to a dominant NDI [204]. Indeed, analysis of the immunoprecipitates revealed that AQP2-insA and WT AQP2 form hetero-oligomers, but, upon co-expression, the WT AQP2 is not able to reach the apical plasma membrane and is directed along with AQP2-insA to the basolateral plasma membrane of MDCK cells.

The strongest evidence of hetero-oligomerization among the mammalian AQPs comes from AQP4. This protein is translated as two major polypeptides, known as AQP4-M1 and AQP4-M23 variants, and it is possible to detect heterotetramers composed of both of them in the plasma membrane [276]. Interestingly, AQP4-M1 and AQP4-M23 as homo- or heterotetramers are able to aggregate into higher–order oligomers called orthogonal arrays of particles (OAPs) [277,278,279]. However, the functional involvement of OAPs clustering is still not completely understood. Supramolecular structures can also be formed by AQP0 but, in this case, the interaction occurs between homotetramers from adjacent plasma membranes, which assemble as octamers, and through specific interactions between loops A and C, forming membrane junctions [31,248].

While the above described cases of hetero-oligomers of mammalian AQPs resulted from the assembly of variants of the same isoform, hetero-oligomers composed of two different AQP isoforms have also been suggested. While a co-localization of AQP5 and AQP2 in Dot1lAC kidneys (mice with Dot1l deficiency in renal Aqp2-expressing cells) is observed by immunofluorescence, co-immunoprecipitation of the GFP-AQP2 and FLAG-AQP5 co-expressed in 293T cells confirms the physical interaction [280]. Interestingly, human AQP5/AQP2 hetero-oligomers are partially retained in the ER/Golgi [280]. A weak physical interaction between AQP2 and AQP0 is also detected using bimolecular fluorescence complementation assays [281] and confirmed by affinity chromatography purification. Despite these interesting data, it cannot be established whether mammal AQPs oligomerize as heterotetramers or assemble in octamers [281].

In conclusion, heterotetramerization seems to be a relevant regulatory process by which cells regulate their transport specificity and trafficking. Taking into account the large number of possible combinations, studying this process could reveal new insights into how cells adapt to their environment. Even so, it was already shown that this is not a general rule for all AQPs.

### 4.3. Gating

The characterization of spinach PIP2;1 structure in open and closed conformations allowed the understanding of the regulatory mechanisms controlling the gating of AQPs [216] and, therefore, the control of the flux through the channel according to the demands. Two main types of AQP pore gating have been proposed: capping or pinching [282]. According to the PIP2;1 model, in a closed conformation, the Leu197 located in loop D together with the residues His99, Val104, and Leu108 create a hydrophobic barrier and serve as a cap of the channel entrance. An opening of the gate is observed after phosphorylation of the Ser115 and Ser274, resulting in a loop D conformation change [216]. Furthermore, divalent cations, such as Ca^2+^, play a role in gating by anchoring the loop D onto the N-terminus, through ionic interactions and hydrogen bonds. In this scenario, phosphorylation of Ser115 disrupts this network of stable interactions between them, leading to the channel aperture. Another regulatory component of the gating is the protonation of the pH sensitive His193 located in the loop D. In acidic environments, protonation of the His193 induces the channel closure [216]. In plants, the acidification of cytoplasm is a complex event associated with drought and flooding, driving anoxia stress responses [283]. In mammals, the mechanism of AQP regulation by pH is not as clear as for plants, and no general mechanism can be proposed as it seems dependent on the AQP subtypes. To illustrate this, Nemeth-Cahalan et al. [284] demonstrated that the external His residues located in loop A and C of the bovine AQP0 could play a dual role according to their acid or alkalization status, leading respectively to less or more water flow. Tornroth-Horsefield et al. [285] suggested a pinching mechanism for AQP0, in which small conformational changes due to protonation pinch the constriction region, limiting the passage of molecules. This mechanism has been proposed since AQP0 water permeability is very low compared to other AQPs, and its cytoplasmic entrance seems to be blocked by a tyrosine residue. This is also probably true for AQP4, although parts of the N- and C-termini are missing in both crystals. The phosphorylation in mammals is generally associated with trafficking, thus leading to an indirect increase in solute transport (see Trafficking section). However, for AQP4, the phosphorylation of Ser180 causes its activation [286,287]. While gating of AQPs is observed in plants and mammals and constitutes a rapid way to control the membrane permeability, it seems that the molecular mechanisms leading to this important regulation have evolved differently according to the kingdom and even the isoforms.

## 5. Conclusions

Plant and mammal AQPs are essential channels facilitating and controlling the diffusion of water and small solutes across the cell membranes. To achieve this fundamental physiological process, the basic function of AQPs, as channels, has been highly conserved during evolution, from bacteria to multicellular organisms. However, when comparing plant and mammal AQPs, several differences are observed. For instance, a huge expansion in the total number of isoforms is detected in plants, and is attributed to the sessile lifestyle of these organisms. Plants have to tightly control water uptake from the soil and its movement across the plant body in ever-changing environmental conditions, a vital physiological process in which AQPs play essential roles in specific cell types and tissues. AQPs are also involved in the diffusion of many different small molecules, some of them, like boron or CO_2_, being essential to plant structure or organic compound synthesis, respectively. Some interesting differences between plant and mammal AQPs come from comparison of the post-translational regulation processes. For instance, the tight regulation of AQP2 trafficking and shuttling between internal vesicles and the plasma membrane controlled by the peptide hormone vasopressin is specific to mammal kidney collecting ducts. In plants, the trafficking of most PIP1s to the plasma membrane is dependent on their heteromerization with the PIP2 proteins. These examples show that, since the divergence of plants and animals, specific regulation processes affecting AQP function have evolved to a better adaptation to the organism development and physiology. However, to achieve these specific regulation processes, common mechanisms, such as phosphorylation or interaction with similar cellular components (i.e., SNAREs), are observed for both kingdoms. Many physiological functions and regulation processes will still have to be discovered and characterized in both plants and mammals. In this context, a constant interchange between the plant and mammal AQP research communities can definitely boost the field and lead to important mutual benefits.

## Figures and Tables

**Table 1:** Functional properties of plant and mammalian aquaporins (AQPs). The substrates transported by plant and mammalian AQPs are shown in following table: H_2_O, water; H_2_O_2_, Hydrogen peroxide; CO_2_, carbon dioxide; Urea; NH_3_, ammonia; Gly, glycerol; As, arsenic; Sb, antimony; Si, silicon; Ions; LA, lactic acid; NO, Nitric oxide; Se, selenium; O_2_, oxygen; Polyols; Bo, boric acid; Purines; Pyrimidines; Carbamides and also corresponding references. PIPs, plasma membrane intrinsic proteins; TIPs, tonoplast intrinsic proteins; NIPs, noduline26-like intrinsic proteins; SIPs, small basic intrinsic proteins and XIPs, uncharacterized X intrinsic proteins.

**Plant AQPs**	**Functional Properties**																			**References**
	**H_2_O**	**H_2_O_2_**	**CO_2_**	**Urea**	**NH_3_**	**Gly**	**As**	**Sb**	**Si**	**Ions**	**LA**	**NO**	**Se**	**O_2_**	**Polyols**	**Bo**	**Purines**	**Pyrimidines**	**Carbamides**	
PIPs	+	+	+	−	−	−	±	NT	NT	+	NT	NT	NT	+	NT	−	NT	NT	NT	[48,49,50,51,52]
TIPs	+	+	−	+	+	+	±	−	−	NT	NT	NT	NT	NT	NT	−	NT	NT	NT	[53,54,55,56]
NIPs	+	−	+	+	−	+	+	+	+	±	+	NT	+	NT	NT	+	NT	NT	NT	[57,58,59,60,61,62,63,64,65,66]
SIPs	+	+	−	−	−	−	−	−	−	NT	NT	NT	NT	NT	NT	−	NT	NT	NT	[67,68]
XIPs	+	+	−	+	+	+	+	−	−	NT	NT	NT	NT	NT	NT	+	NT	NT	NT	[69,70,71,72]
**Mammal AQPs**																				
	**H_2_O**	**H_2_O_2_**	**CO_2_**	**Urea**	**NH_3_**	**Gly**	**As**	**Sb**	**Si**	**Ions**	**LA**	**NO**	**Se**	**O_2_**	**Polyols**	**Bo**	**Purines**	**Pyrimidines**	**Carbamides**	
AQP0	+	−	+	−	−	−	−	−	−	+	NT	NT	NT	NT	NT	NT	NT	NT	NT	[73,74,75,76]
AQP1	+	+	+	−	+	−	−	−	−	+	NT	+	NT	NT	NT	NT	NT	NT	NT	[75,77,78,79,80,81,82,83,84,85,86,87,88,89,90,91,92]
AQP2	+	−	−	−	−	−	−	−	−	NT	NT	NT	NT	NT	NT	NT	NT	NT	NT	[75,93,94]
AQP4	+	−	+	−	−	−	−	−	−	NT	NT	NT	NT	NT	NT	NT	NT	NT	NT	[27,75,84,86,95,96,97,98,99,100]
AQP5	+	−	+	−	−	−	−	−	−	NT	NT	NT	NT	NT	NT	NT	NT	NT	NT	[75,82,90,101]
AQP6	+	−	+	+	+	+	−	−	−	+	NT	NT	NT	NT	NT	NT	NT	NT	NT	[75,82,90,102]
AQP8	+	+	−	+	+	+	−	−	−	NT	NT	NT	NT	NT	NT	NT	NT	NT	NT	[84,98,103,104]
AQP3	+	−	−	+	−	+	+	+	+	NT	NT	NT	NT	NT	+	NT	NT	NT	NT	[27,90,105,106,107]
AQP7	+	−	−	+	+	+	+	+	+	NT	NT	NT	NT	NT	NT	NT	NT	NT	NT	[25,27,84,90,108,109]
AQP9	+	−	+	+	+	+	+	+	+	NT	+	NT	NT	NT	+	NT	+	+	+	[27,84,90,109,110,111,112,113,114]
AQP10	+	−	−	+	−	+	+	+	+	NT	NT	NT	NT	NT	NT	NT	NT	NT	NT	[25,26,27,84,90,98,109,115,116,117]
AQP11	+	−	−	−	−	+	−	−	−	NT	NT	NT	NT	NT	NT	NT	NT	NT	NT	[118,119]
AQP12A	NT	NT	NT	NT	NT	NT	NT	NT	NT	NT	NT	NT	NT	NT	NT	NT	NT	NT	NT	-
AQP12B	NT	NT	NT	NT	NT	NT	NT	NT	NT	NT	NT	NT	NT	NT	NT	NT	NT	NT	NT	-
AQP13	+	−	−	+	−	+	−	−	−	NT	NT	NT	NT	NT	NT	NT	NT	NT	NT	[120]

Here: +, transport; −, not transport; ±, controversial; NT, not yet tested; -, no reference found.

## Abbreviations

AQP	Aquaporin
MIP	Major intrinsic protein
TM	Transmembrane α-helice
GLP	Aquaglyceroporin
HGT	Homologous gene transfer
PIP	Plasma membrane intrinsic protein
TIP	Tonoplast intrinsic protein
NIP	NOD26-like intrinsic protein
SIP	Small intrinsic protein
XIP	Uncharacterized X intrinsic protein
GIP	GLpF-like intrinsic proteins
LIP	Large intrinsic proteins
ER	Endoplasmic reticulum
NDI	Nephrogenic diabetes insipidus
SNARE	Soluble *N*-ethylmaleimide-sensitive factor protein attachment protein receptor
SNAP	Synaptosomal-associated protein
VAMP	Vesicle-associated membrane proteins
TSPO	Tryptophan-rich sensory protein/translocator
LIP5	Lysosomal Trafficking Regulator-interacting protein 5
MVB	Multi-vesicular body
P_f_	Osmotic water permeability coefficient
OAP	Orthogonal arrays of particles
